# Editorial: Mindfulness and health

**DOI:** 10.3389/fpsyg.2023.1137730

**Published:** 2023-02-03

**Authors:** Shuang Lu, Chien-Chung Huang, Brian Hall, Marcelo Demarzo, Ulrich Kirk

**Affiliations:** ^1^School of Social Work, University of Central Florida, Orlando, FL, United States; ^2^School of Social Work, Rutgers, The State University of New Jersey, New Brunswick, NJ, United States; ^3^Center for Global Health Equity, New York University Shanghai, Shanghai, China; ^4^Mente Aberta - The Brazilian Center for Mindfulness and Health Promotion, Department of Preventive Medicine, Universidade Federal de São Paulo, São Paulo, Brazil; ^5^Department of Psychology, University of Southern Denmark, Odense, Denmark

**Keywords:** mindfulness, health, social support, burnout, intervention

Mindfulness has positive effects on physical and mental health. However, less is known about the antecedents to mindfulness, the mechanisms of mindfulness on health, and the cost and effectiveness of mindfulness-based interventions on health outcomes. This Research Topic brings research and empirical attention to antecedents to and mechanisms of mindfulness on health by including 10 contributions from authors in diverse settings and populations globally.

For instance, in the study by Xie et al. of Chinese social workers' burnout, mindfulness increased with perceived social support and in turn reduced job burnout, while mindfulness played a modest mediating effect. In another study of Chinese food and package delivery drivers' burnout (Zhang et al.), mindfulness reduced the positive relationship between job demands and drivers' burnout while it exacerbated the negative relationship between job resources and burnout. In other words, participants' levels of mindfulness acted as a protective factor from high job demands as well as a promotive factor for utilizing job resources.

Also worth noting is the increasing emphasis on the impact of mindfulness on biological processes. In Ng et al. study of a mindfulness-based stress reduction (MBSR) program in Taiwan, body scan was associated with declines in electroencephalogram (EEG) spectral powers in comparison with mindful breathing exercises; the MBSR group's improvement in emotion regulation was also associated with the EEG spectral changes in the theta, alpha, and low-beta bands. In another example, Grupe et al. study among police officers in the U.S. showed lower cortisol awakening responses among participants following an 8-week mindfulness intervention (but no effects for inflammatory biomarkers), along with improvements in distress, mental health, and sleep using self-reported surveys.

However, the complexity of using biomarkers inevitably adds another layer of difficulty in data collection and analysis. For example, Ng et al. study that used simultaneous EEG-functional magnetic resonance imaging (fMRI) signal data had a small sample size (*n* = 17 in the treatment group and 14 in waitlist control group) due to limited MRI scanning sessions. Given the nuance in operationalizing biological measurements (e.g., frequency range of EEG), the results also warrant cautious interpretation when comparing the biomarkers across studies. In the study of Grupe et al., in-person assessments could only be conducted in participants' non-work days, which led to lengthy post-intervention and follow-up assessments over several weeks. Future research that aims to collect biological data should take these limitations into consideration.

Several articles in this Research Topic focus on under-studied and under-served populations, such as delivery workers, police officers, and teachers in special education schools. As Grupe et al. mentioned, the strength of their study was their sustained engagement with marginalized communities, which allowed them to tailor their intervention for community members and prioritize the needs of participants. Although existing mindfulness literature focuses less on marginalized populations, future research should involve these communities throughout the research process, from study planning to outcome evaluation, to maximize intervention effectiveness for individuals and families faced with greater challenges.

Some studies focus on previously under-examined outcomes or measurement issues. Cheung et al. examined the effect of mindfulness on grit, as measured by perseverance of effort and consistency of interests, factoring in participants' adverse childhood experiences among Chinese college students. Their results indicated a protective effect: Mindfulness was positively related to students' grit, and the negative effects of adverse childhood experience on grit became non-significant when controlling for level of mindfulness. Malakoutikhah et al. cross-sectional study compared the association between two different measures of mindfulness (the Relaxation/Meditation/Mindfulness Tracker t-Persian version [RMMt-P] vs. the Freiburg Mindfulness Inventory-Short-Form-Persian version [FMI-P]) and participants' anxiety, anger, and general health among an Iranian non-clinical adult sample. Their study suggests a “broadband” mindfulness measure (RMMt-P, which includes various mindfulness states such as thankfulness, relaxed, pleasant, and spaciousness) was a better predictor of general health and anger than a “narrowband” measure (FMI-P, a unidimensional measure of mindful presence and acceptance). Further, they suggest only some, but not all, mindfulness states had better predictive strength (e.g., mindful love, thankfulness, transcendence). Considering the complexity of conceptualizing and operationalizing “mindfulness,” future research should consider how the measurement itself may make a difference in the results. This “measurement effect” may also vary by age, education level, and recruitment setting (e.g., clinical vs. non-clinical sample).

However, both above-mentioned studies used cross-sectional, self-reported surveys that cannot lead to a causal conclusion. This limitation may be addressed by more longitudinal studies and experimental designs. One example is the 3-wave longitudinal study by Su et al., who found that mindfulness mediates the relationship between positive parenting and Chinese middle school students' maladaptive psychological outcomes. As acknowledged by Su et al., although their multi-wave surveys allowed more rigorous mediation analysis than cross-sectional studies, further experimental designs are needed to rule out other potential factors (e.g., adolescents' resilience) and monitor the mediating effects over longer period of time.

In addition to outcome measures, baseline measures are also relevant to intervention effectiveness. In Vergara et al. comparison of three prior randomized controlled trials, participants' baseline level of mindfulness showed significant impact on individual trajectories in the intervention. Individuals who started with low-level mindfulness may benefit differently from those started with a higher level. Their findings necessitate the measurement of participants' initial status (such as the relatively stable mindfulness trait) in intervention planning. Whether a standard mindfulness intervention program works equally for everyone in the group is also worth further investigation. Echoing their conclusions, Hu et al. meta-analysis of mindfulness on empathy among healthy adults suggested that although overall mindfulness-based interventions showed a positive effect on empathy (*d* ranged 0.164–0.579), the effects varied by intervention dosage, format (online vs. offline), and program type.

In conclusion, this Research Topic exemplifies several potential future directions of mindfulness research in health-related fields: increasing interdisciplinary collaborations; integration of biological, emotional, and behavioral processes; interpretation of mindfulness practices in different cultural contexts; and a shift of focus from efficacy testing to moderating and mediating mechanisms of mindfulness-based interventions. With several decades of emerging empirical research, we expect there will be increase in rigorous research rooted in marginalized communities, adapted to unique cultural contexts, and that crosses disciplinary boundaries.

## Author contributions

All authors listed have made a substantial, direct, and intellectual contribution to the work and approved it for publication.

